# Supplying the Power Requirements to a Sensor Network Using Radio Frequency Power Transfer

**DOI:** 10.3390/s120708571

**Published:** 2012-06-26

**Authors:** Steven Percy, Chris Knight, Francis Cooray, Ken Smart

**Affiliations:** 1 Commonwealth Industrial Research Organisation (CSIRO), P.O. Box 330, Newcastle, NSW 2300, Australia; E-Mail: steven.percy@csiro.au; 2 Commonwealth Scientific and Industrial Research Organisation (CSIRO), P.O. Box 76, Epping, NSW 1710, Australia; E-Mails: crfsc@optusnet.com.au (F.C.); ken.smart@csiro.au (K.S.)

**Keywords:** radio, supercapacitor, wireless power, track, position, energy scavenging, energy transfer, impedance matching

## Abstract

Wireless power transmission is a method of supplying power to small electronic devices when there is no wired connection. One way to increase the range of these systems is to use a directional transmitting antenna, the problem with this approach is that power can only be transmitted through a narrow beam and directly forward, requiring the transmitter to always be aligned with the sensor node position. The work outlined in this article describes the design and testing of an autonomous radio frequency power transfer system that is capable of rotating the base transmitter to track the position of sensor nodes and transferring power to that sensor node. The system's base station monitors the node's energy levels and forms a charge queue to plan charging order and maintain energy levels of the nodes. Results show a radio frequency harvesting circuit with a measured S11 value of −31.5 dB and a conversion efficiency of 39.1%. Simulation and experimentation verified the level of power transfer and efficiency. The results of this work show a small network of three nodes with different storage types powered by a central base node.

## 1. Introduction

As wireless sensor technology develops, there are new applications being found causing networks to be situated in locations where direct connection or easy access to the node is not possible. Examples of these locations include rainforest regeneration monitoring under dense tree foliage, inside buildings for comfort sensing, in mineshafts for safety monitoring, inside enclosed structures for structural monitoring or attached to animals for position and health monitoring [1]. Usually inaccessible sensor nodes are fitted with large capacity batteries that can supply power for the nominated lifetime of the sensor node. This can become impractical where size, weight and long operation are important for the sensor network. One solution to this problem is by harvesting ambient energy to supply or extend the sensor nodes' lifetime. However, power will have to be supplied remotely when there is no source of ambient energy at the location of the sensor node. This has led to research into power transmission through ultra high frequency (UHF) radio waves.

The use of radio waves for power transfer is not a new concept. Radio Frequency (RF) power transmission dates back to experiments by Heinrich Hertz in 1886, where he demonstrated that by using two similar antennas, one to transmit and one to receive, radio waves could propagate through free space [2]. Throughout the past 50 years as amplifier, antenna and rectification technologies advanced, research has been applied to high power RF transfer; Brown [2] gives a detailed discussion of this early work. Included in this discussion was work on an airborne microwave powered platform and development of a theoretical approach to convert solar radiation on an orbiting satellite to ground based electrical power [3].

UHF wireless power transfer is also used in radio frequency identification (RFID) [4,5]. In the normal operation of these devices, a RFID reader transmits the UHF signal and a receiving antenna connected to a Dickson voltage multiplier converts the UHF signal to a DC voltage capable of powering up a logic circuit. The logic circuit controls a phase modulator circuit to modulate a small amount of information into the backscattered signal.

In recent times, powering of wireless sensors or small electronic devices using radio frequency power has been the focus of most researchers. A great deal of this research aims to harvest radio frequency energy from mobile phone towers, television station or Wi-Fi transmitters; examples of these projects include [6,7]. One problem with this approach is the associated path loss causing a low power density at the receiving point. In this situation, it is possible to receive a usable power if the equivalent receiving area of the antenna is large enough. There are two slightly different approaches to this; the first is the use of multiple antennas and rectifiers and series connection of the harvesting circuits at the DC side [6]; the second uses a high gain antenna such as a Log-Periodic or Yagi-Uda (Yagi) antenna [7]. The problem with these approaches is that the receiving antennas become large, heavy and are required to be aligned to the RF source. This limits many of the applications where size, weight and the need to be mobile are important, an example being animal health monitoring.

In [7] and described in further detail in [8], is the testing and design of a wireless power transfer node that uses a commercially available RFID reader to charge a capacitor to power a microcontroller. The microcontroller will read the sensor data and modulate the information into the back scattered signal in the format that the RFID reader will recognize. These devices are capable of providing sensor information in some of the situations defined in the first paragraph, although when higher processing power or sensor network functionality is required this solution might not be viable. The noted system, in its current configuration, does not apply autonomous control to decide when or where the RF power is supplied. In order for larger number or higher-powered nodes to operate improvements have to be made, this along with other improvements will be addressed.

Described within this article is a system that will maintain the energy levels of a close range network of sensor nodes by autonomously tracking the position of sensor nodes and supply power to that sensor node. Discussed is the design of the harvesting circuit responsible for capturing the RF energy. An optimum AC to DC conversion efficiency was achieved by selecting an appropriate storage voltage and received RF power for the circuit design. This circuit uses an omni-directional power-receiving antenna allowing it to be mobile within the network. Included in a detailed discussion is the protocol that makes the tracking function possible. Results will be given showing three sensor nodes with different storage technologies for comparison and demonstration of the functionally of the system.

## 2. Wireless Power Transfer System

**Figure 1.**
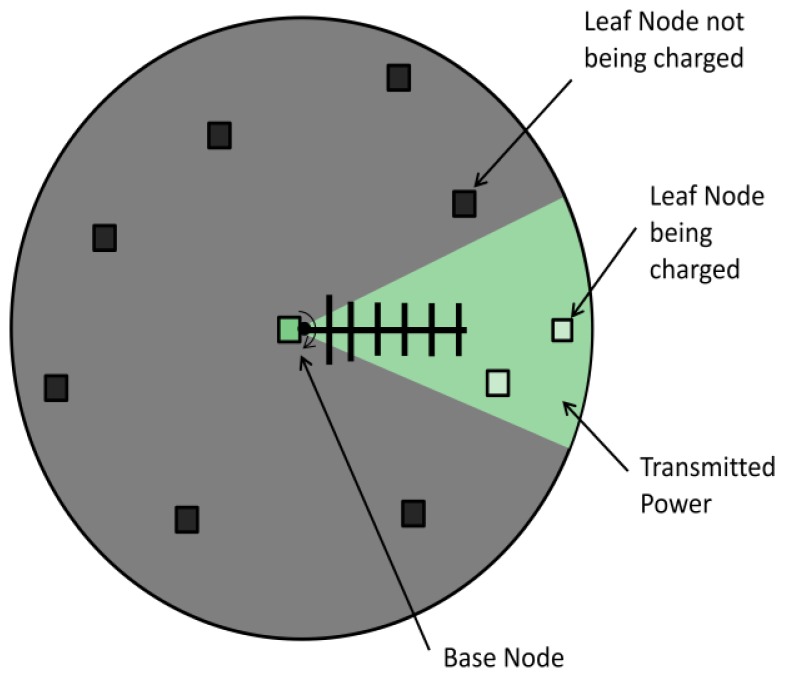
The RF transfer system setup.

There are two node classifications used in the system; leaf nodes are responsible for measuring the sensor information (*i.e.*, temperature), and the base node which is responsible for supplying power to the leaf nodes, managing the energy of the sensor network and logging the sensor data. A cluster of leaf nodes surrounds the base node as shown in Figure 1. The base node controls the azimuth of the transmitting antenna to direct RF power to the receiving node. When a leaf node in the network requires power, it issues a request to the base node, which begins a seek protocol when available for charging. To find a leaf sensor node the protocol rotates the transmitter until the leaf node responds to the base indicating that sufficient power is received. If no nodes in the network require power, the base node shuts down the transmitter to conserve power; more details about this operation are given below. The base and leaf sensor nodes used in this experiment are CSIRO Fleck™3B wireless sensors. These consist of an ATmega128L microcontroller, Nordic RF 905 radio chip, an onboard temperature sensor and connectors for sensor daughter boards; see references [10,11] for more details.

### 2.1. Design of the Leaf Nodes

**Figure 2.**
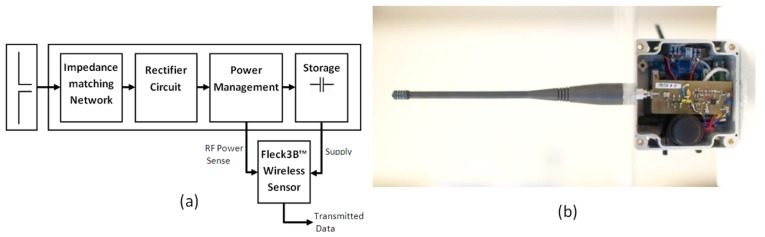
The leaf node: (**a**) harvesting circuit block diagram and (**b**) showing the power receiving antenna, harvesting circuit and Fleck™3B sensor.

The design for the harvester circuitry is based around a similar architecture to that used in [4–7]. This involves the use of an impedance-tuned rectifier circuit to convert the received RF power into a useable DC voltage. Figure 2(a) shows the modules which make up the leaf node's circuit, this consists of a secondary 50 Ω omni-directional receiving antenna (433 MHz), a high pass impedance matching network, rectifier circuitry, power management, energy storage and a Fleck™3B wireless sensor. The rectifier circuitry selected for this application was a two-stage Dickson voltage multiplier. Karthaus *et al.* [5] discusses the benefits of this topology. It also exhibits a clean and simple circuit layout and a manageable impedance change. The harvesting circuit's impedance changes because of different received powers, due to different separation distances and storage device voltages as the battery or supercapacitor charges.

Generally, RFID power harvesting circuits such as described in [4,5] only need to produce a voltage sufficient to power up a logic circuit and operate efficiently at low RF power (10–100 μW). Conversely, for this application to ensure that continual charging is not needed a much higher received power and different operating voltage range was required so that energy can be stored for later use. The critical component that allows the circuit to operate efficiently to these constrains is the design of the circuit's impedance matching network. An RF input power and storage device voltage had to be selected for the design of this impedance matching network. For this experiment 6 dBm (4 mW) RF input power was selected as a ‘nominal power’ that the system could provide. As a minimum operating point for the sensor nodes a storage device voltage of 2.5 V was selected as this is the minimum voltage the node will operate at without switching off. The results section will verify the quality of the impedance match as the storage device charges.

To design the inductive and capacitive high pass impedance matching network to these constraints, the series component of the matching network was short-circuited and the shunt component was removed. A network analyser was used to measure the input impedance of the harvesting circuit, this had to be set to a higher input power than the matching network was being designed to operate. Due to the impedance mismatch, the actual power level entering the circuit was lower than the network analyser setting due to the high percentage of RF power reflected. A Smith chart was used to determine the lumped component values that provide a 50 Ω impedance match allowing the maximum RF power to be rectified.

The power management block in Figure 2 performs two functions: it protects the storage device from being over charged and provides a voltage level, which the wireless sensor reads to determine whether RF power is being received at an appropriate level.

For this experiment, omni-directional receiving antennas were used for the leaf nodes. This allows the nodes to be scatted around the base transmitter without concern for the directional gain of these antennas. This is an additional antenna to the onboard antenna used by the FLECK™3B wireless sensor. This assists in making the leaf nodes mobile. The use of a higher gain receiving antenna could increase the received power, but this would necessitate careful alignment of the leaf nodes to the base transmitter.

For the system to track the sensor node position, it is critical for the leaf node to sense the level of received RF power at its location. The sense voltage from the power management circuit indicates this, as the higher the received power the higher this voltage will be, Figure 2(a) shows this connection. The Fleck™3B wireless sensor monitors the sense voltage and its own storage device voltage using its analog to digital converter. The sensor can make an assessment whether sufficient power is being received to charge its battery if this voltage meets the condition in Equation (1):

(1) $${\text{V}}_{\text{sense}}>{\text{V}}_{\text{B}}+{\text{V}}_{\text{F}}$$

In this expression V_B_ is the storage device voltage, V_F_ is the forward voltage of the diode used in the circuit (0.7 V in this case). A diode with a very low reverse leakage is required so that the leakage current does not give a false reading for V_sense_, and result in unwanted discharge of the storage device.

### 2.2. Leaf Node Configurations

Charging of three leaf nodes each with different storage types is included to compare the effects of storage on node operation. The storage types used were two different commercially available supercapacitors and a low capacity commercially available NiMH battery; these three configurations are summarised in Table 1. To halve the equivalent series resistance of the capacitor, two identical supercapacitors were connected in parallel. If this was not done the high series resistance of the Panasonic supercapacitor (100 Ω) caused a significant voltage drop during the relatively high current draws needed for sensor data transmissions. This reduces the operating voltage range of the capacitor and as a result reduces the available energy. Two voltage thresholds were set: the lower voltage threshold, used to indicate when charging is required, and the upper voltage threshold, used to indicate when charge is complete. The selection of these thresholds can be such that it encourages the battery or supercapacitor to be cycled to maximise its cycle lifetime. The thresholds in Table 1 Table 1 were selected to eliminate overcharging of the storage device and show regular charge cycles. When the node is waiting for a charge, the lower threshold must be high enough that the stored energy does not deplete while in the charging queue. The separation distances shown in Table 1 were used to demonstrate different received power levels and consequently different charging times.

### 2.3. Design of the Base Node

**Figure 3.**
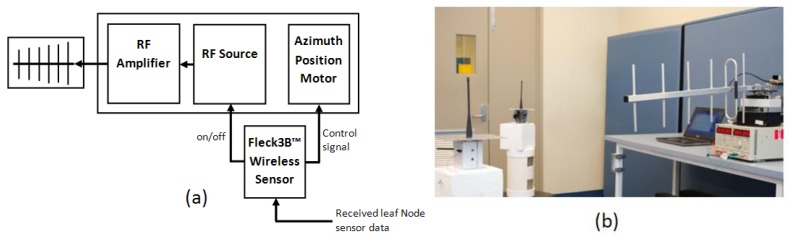
The base node: (**a**) power transmitter circuit and (**b**) the experimental setup showing the base node, the transmitting antenna, the rotator, and two leaf nodes.

The base node uses the transmitter structure outlined in Figure 3(a). The function of this design is to autonomously direct RF power to the location of the leaf nodes. A low power commercially available transmitter oscillator with no modulation was used to generate the 433 MHz signal. This signal was increased using an RF amplifier, and the voltage supplies were adjusted to give 20 dBm (100 mW) output power from the RF amplifier. This RF power was transmitted outward using a commercially available directional Yagi antenna.

One Fleck™3B wireless sensor performs several control functions for the base node. It produces a pulse width modulated control signal to set the azimuth position of the 360° motor that rotates the base node and antenna. It also performs some processing: calculating the angle of rotation based on the received data from the leaf nodes, and managing the leaf node charging order. It switches on and off the base transmitting antenna to conserve power if no node in the network requires charging or is in range.

The base node and transmitter in this experiment was supplied from a desktop DC power supply, this could be replaced with another form of energy harvesting such as solar. Shown in Figure 3(b) is the base, powering one of two visible leaf nodes.

### 2.4. Programming of the Sensor Network

**Figure 4.**
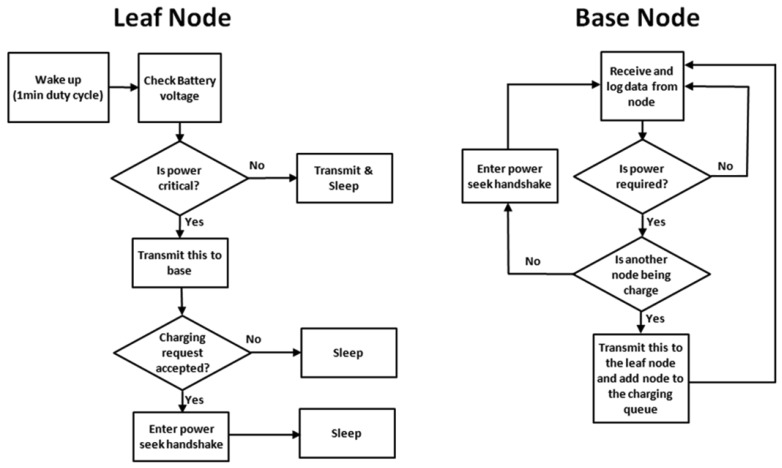
The programming process flow diagrams used for the leaf and base node.

Figure 4 shows the programming process flow for the leaf and base nodes. Multithread programming allows the single base node to receive and log data from the leaf nodes in the network. The base node performs a variety of functions according to the data packet received from the leaf nodes.

All the leaf nodes wake up on 1-minute intervals. Their wake-up schedules are staggered to prevent multiple leaf nodes from waking up at the same time. Upon waking up, the node checks its battery voltage; if this is critically low, the node will raise a flag in its normal transmission to indicate to the base node that charging is required; it then waits for a response from the base node. The base node will respond with a transmission to indicate whether the charging request has been accepted or denied. Charging is denied if another node is already receiving a charge.

If charging is denied the base node will add the leaf node's identification number to a charging queue and the leaf node will go back to sleep. If charging is accepted the base and leaf nodes will enter a seek protocol, which locates the azimuth position of the leaf node so that power can be supplied in that direction. The seek protocol operates through a cycle of base-to-leaf and leaf-to-base transmissions. The azimuth angle of the base node antenna is incremented by 7 degrees each cycle, corresponding to a fraction of the beam width of the transmitting antenna. As it does this power is only directed outward into a single azimuth sector. The protocol continues, incrementing the azimuth position, until the base node receives the strongest leaf node response, indicated by the condition given in Equation (1). Then the base node antenna remains stationary until the storage device reaches its upper trigger voltage as indicated in Table 1. The leaf node then enters its sleep mode to conserve power, still waking up on its regular 1 minute duty cycles to transmit its sensor data.

To improve the next seeking efficiency, the base node logs the azimuth angle of leaf nodes it has found to its memory. The next time a charging request is issued by a previously charged leaf node, the base node antenna can be rotated directly to the correct azimuth angle, reducing the time and energy required for the seek cycle. If the leaf node is stationary, charging will then begin straight away. If it is a mobile node and moved since the last charge, it will enter the seek protocol allowing its new location to be found starting from its last known location.

### 2.5. Multiple Node Power Transfer Requirements

To identify whether the system is capable of supplying the power requirements to a sensor network it was important to quantify how much power is required to operate the leaf nodes in their different operational states. Because the leaf nodes performed no other functions for this experiment, there were only two states to consider; requiring or not requiring a charge. The average power was determined by measuring the current, i(t), through a highly accurate, low-value resistor in series with the power source. This was repeated for both 3.5 V and 4 V supply voltage, v(t). A time integral of the instantaneous power consumption, P(t) = v(t)i(t), was calculated to give the energy required to supply the leaf node during the one minute duty cycle between transmissions. This was divided by the cycle time T to give the average power P_av_ required to run the leaf node, as shown in Equation (2). By increasing the cycle time, it is possible to reduce the average power required to run the sensor network, although as shown by McGarry [9] a limit will be approached as the energy required while sleeping makes up the majority of the energy usage of the leaf node per cycle for any practical cycle time:

(2) $${\text{P}}_{\text{av}}=\frac{1}{\text{T}}\int_{0}^{\text{T}}\text{v}(\text{t})\text{i}(\text{t})\text{dt}$$

Once the power consumption of the leaf node is known it can be compared to the maximum power that can be harvested P_her_ for a given leaf node position, and the time available to supply power to this leaf node as a member of the network. To describe this, a charge intermittency factor μ defined by Equation (3) as the ratio of the time t_c_, available for charging a leaf node to the total time t available for all nodes to be charged. This implies Equation (4) because all nodes must share the available time:

(3) $$\mu =\frac{{\text{t}}_{\text{c}}}{\text{t}}$$

(4) $$\mu_{\text{node}1}+\mu_{\text{node}2}+\cdots +\mu_{\text{node n}}\leq 1$$

Equation (5) then calculates the energy stored in the node after a time t where E_start_ is the initial energy stored in the battery or capacitor. Leakage from the storage device is neglected in Equation (5) but could become significant when P_har_ and P_av_ are low:

(5) $${\text{E}}_{\text{node}}(\text{t})={\text{E}}_{\text{start}}+\mu {\text{P}}_{\text{har}}\text{t}-{\text{P}}_{\text{av}}\text{t}$$

From the definition of Equation (3), the requirement indicating if adequate power is received by a leaf node to allow it to operate as part of the network is defined as:

(6) $${\text{P}}_{\text{har}}\geq \frac{{\text{P}}_{\text{av}}}{\mu }$$

Equations (4) and (6) must both be satisfied to supply the power requirements to a sensor network using the system. If these are not satisfied, the initial energy storage will eventually deplete in one or more leaf nodes, which will consequently power down. For a large multimode network the base node may make a decision to cease charging leaf nodes with low values of P_har_ to give other leaf nodes priority, due to their more efficient charging, and thereby maintain the energy level of the complete network. The results reported below will clarify this analysis.

The leaf nodes require power to allow for the seek protocol to operate; if E_node_ (t) is zero on system start up or if power has depleted while charging another node this node will not be able to be found. To deal with this in the case where a node has not moved since the last charge and when no nodes require power the base could return to the last known position with an aim to ‘jump start’ that sensor again; in the current experiment this is not applied.

### 2.6. Verifying the RF Performance

**Figure 5.**
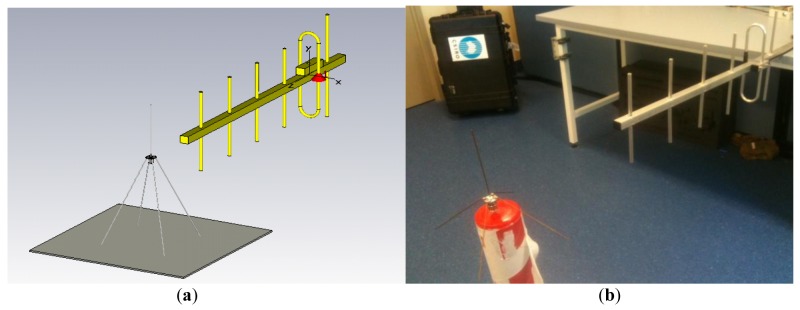
(**a**) The Microwave Studio model of the Yagi antenna and the whip antenna separated by 90 cm; (**b**) simulation verification in the laboratory.

The antennas are operating in the near field, so equations for far field path loss cannot be used to determine the RF power level that can be received by the leaf-node antennas, and would also ignore the effect that mutual coupling will have on the power transfer. Instead, the antenna arrangement was modeled using CST Microwave Studio and a two-ray propagation model [15,16]. Figure 5(a) shows this 3D model.

The antennas are operating in the near field, so equations for far field path loss cannot be used to determine the RF power level that can be received by the leaf-node antennas, and would also ignore the effect that mutual coupling will have on the power transfer. Instead, the antenna arrangement was modeled using CST Microwave Studio and a two-ray propagation model [15,16].

The received power level was determined for different separation distances. The CST Microwave Studio analysis models the mutual coupling between the two antennas, and the two-ray propagation model accounts for the effect of reflections from the ground. In this simulation, as in the experimental setup, the antennas where located 1.7 m above the ground. It was assumed that the complex relative permittivity of a typical ground of this type is given by ε_rc_ = ε_r_ – j60σλ, where ε_r_ is the real part of the dielectric constant of the ground relative to free space, σ is the conductivity of the ground, and λ is the wavelength of the transmitted signal in meters. Typical constants for concrete are ε_r_ = 15 and σ = 0.005 mΩ/meter [17]. These values, together with the wavelength corresponding to a transmitting frequency of 433 MHz, were used to calculate the Fresnel reflection coefficient for the two-ray model analysis. A simulation of the power transfer for a range of distances up to 3.3 m was performed.

The simulated power transfer was then verified in the laboratory (Figure 5(b)), using an RF power meter to measure the received power at each separation distance; at the same time the value for the power sense voltage was measured. The experiment was repeated for a variation of azimuth angles over 180° at a fixed separation distance of 1 meter to demonstrate the ability to track the position of the node sensor node while the base antenna is rotating in the seek protocol.

An analysis was conducted to verify that the impedance match is maintained between the 50 Ω leaf node receiving antenna and the harvesting circuit as the storage device charges. This was done using a network analyser to charge a single GS 280 Cap-xx supercapacitor at 6 dBm input power at 433 MHz. Measurements at regular time intervals for supercapacitor voltage, reflection coefficient S11, and the circuit input impedance were recorded. This allows a correlation between storage voltage and S11 to ensure the circuit is working efficiently.

To determine the complete AC to DC conversion efficiency, current was measured using a highly accurate current sense resistor placed in series with the storage device. The storage device was charge to 2.5 V and 6 dBm RF input power was supplied to the circuit, the reason these levels were used is detailed in Section 2.1. The power into the storage device was determined by multiplying the measured capacitor charge current by 2.5 V. Calculated power was divided by 4 mW (6 dBm) to determine the conversion efficiency.

The final analysis was to power the three nodes at the different separation distances detailed in Table 1. Each node was situated at 50° azimuth separation from one another; this was outside the beam width of the antenna. During the system operation, the storage element voltage for the three nodes was logged at a 5 kHz sample rate.

## 3. Results and Discussions

### 3.1. Power Transfer and Sense Performance

**Figure 6.**
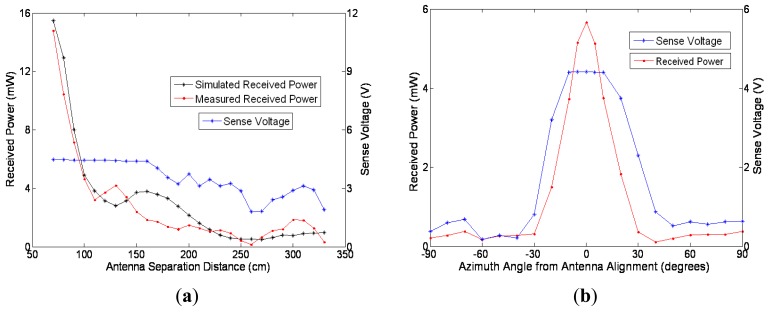
(**a**) Received power and sense voltage as separation distance increases; (**b**) Plot of received power and sense voltage at different azimuth angles for antennas at 1-meter separation.

The plot in Figure 6(a), demonstrates the received power as separation distance increases. This shows good agreement between measured and simulated results. The main difference between the measured (red) and simulated (black) results are the first earth reflection multipath peak being offset by 0.3 meters. This can be attributed to the inability to model the propagation environment exactly, including multi path signals. In addition, there is an inability to include all the minor details of the antennas, especially that of the monopole antenna, into the CST Microwave Studio model. Another result demonstrated here is that the power transmission will operate indoors, which is a possible application for the system, as described as an important application in Section 1. Figure 6(a) also demonstrates and confirms a correlation between received power and sense voltage used for the tracking function. The sense voltage increasing fast until the condition in Equation (1) is met; and a much slower rate after this condition since power is flowing into the storage device.

Figure 6(b) shows the measured received RF power and sense voltage at a separation distance of 1m while the antenna scans from −90° to 90° with the leaf node located at zero degrees. This plot verifies the use of the sense voltage to determine the alignment of the leaf node and the base transmitter. Comparatively to Figure 6(a), it can be noticed the peak of the sense voltage levels off as it meets the condition described in Equation (1).

The characteristic that causes the sense voltage to level off could be eliminated by programming the sensor to disconnect the harvester circuit from the battery during the seek protocol. This would have the effect that this voltage would go much higher when the antennas are aligned. As a result, the leaf nodes could make a more accurate assessment of the received power and hence a more accurate alignment between the antenna and the sensor node, the current system does not apply this.

**Figure 7.**
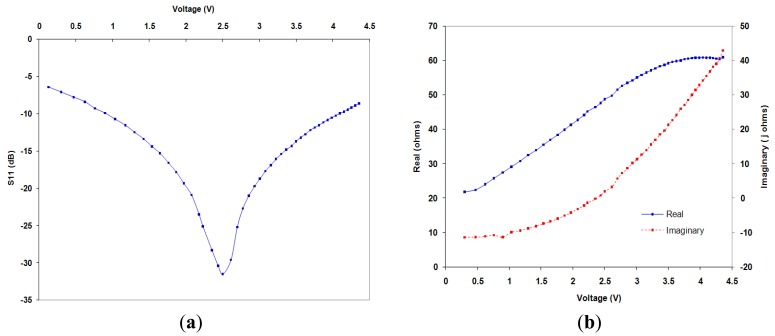
(**a**) change of S11 as supercapacitor charges at 6 dBm; (**b**) Impedance change as supercapacitor charges.

Figure 7(a) demonstrates the measured change in S11 as the storage device charges. It can be seen that S11 reduces when not at the designed voltage due to the circuit impedance change as a result of the different diode currents in the rectifier circuit. Although S11 remains at a low enough level to allow for efficient rectification through this expected charge range. This demonstrates that it would be an advantage to use a storage device that maintains a constant voltage while it charges and discharges; one example would be to cycle a lithium polymer battery around 4.0 to 4.3 V.

Figure 7(b) shows the impedance change of the as the storage voltage increases the imaginary part of the input impedance becomes more inductive (red) and the resistive component increases (blue).

### 3.2. Power Usage and Efficiency

The average power for the 1-minute duty cycle, including the wake up function, transmit and sleep power between transmissions; when above the lower voltage trigger was 0.64 mW and when below the lower trigger level was 0.91 mW. The reason for this difference is the short receive time required to tell the node to sleep during the different mode of operation, as described in Section 2.4.

At the tuned operating parameters of 2.5 V storage device voltage and 6 dBm input power, S11 for the circuit was measured to be −31 dB. In addition, the complete AC to DC conversion efficiency of the harvesting circuit was found to be 39.1%. The substrate loss in the rectifier circuit, and to a lesser extent the impedance mismatch, is contributing to this efficiency. Full wave rectification could improve this, the effect of this on impedance change would need to be analysed. It should also be noted that if the conversion efficiency could be increased the range of the system would also increase.

### 3.3. Sustained Operation

**Figure 8.**
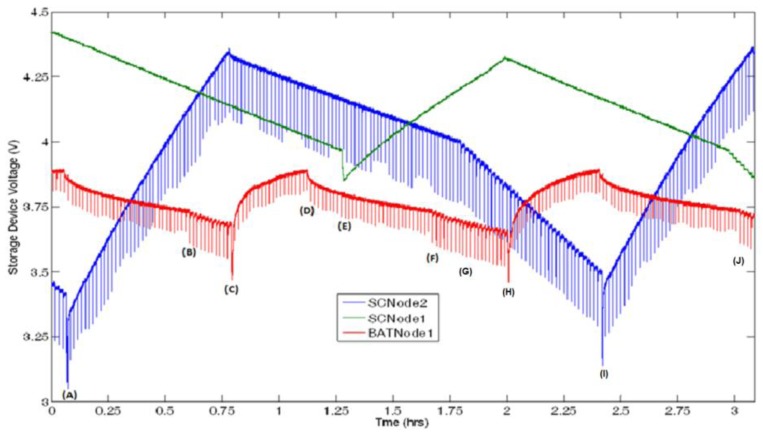
Storage voltage of three nodes being powered from the system.

The following describes the functions of the autonomous radio frequency supply system in operation using Figure 8 as a 3-hour snapshot of the system powering the three nodes and separation distances outlined in Table 1. The marked points highlight the programmed functions as discussed in Section 2.3. The results shown here are characteristic of the system operating, and are consistent for other time segments.

Near the beginning of this time segment marked by (A), BATNode1 (a leaf node powered from a NiMH battery) has reached its upper trigger voltage and ceased charging. Since SCNode2 (a leaf node powered by a Panasonic supercapacitor) is below its lower trigger voltage, it has previously requested charging from the base and is sitting in the charging queue within the program on the base node. It then enters the seek protocol which can be noted by the lower spikes at (A); once found this node begins charging. Midway through the charge, noted by (B), BATNode1 has reached its lower trigger voltage, since SCNode1 is not 100% full, charge is denied. As mentioned in Section 3.2, the power required for the receive operation to tell BATNode1 to go back to sleep is the reason for the increased slope of discharge. When SCNode2 reaches its upper threshold voltage at (C), the base then seeks out BATNode1 until it is found and begins charging. Since this location is stored in the memory from the last seek search, the node is found straight away. If the node had moved since the last charge, it would have been found again and the new location stored in the memory. When BATNode1 reaches its upper trigger voltage at (D) no nodes require charging, at this point the base node shuts down the transmitter. This idles until SCNode1 requests power at (E) where the node is tracked and begins charging. This cycle continues, with these same functions reoccurring at (F–J), maintaining the energy storage levels of all nodes in the network.

A key comparison between the two supercapacitors can be made from Figure 8. Since the Panasonic supercapacitor has a relatively high series resistance compared to the Cap-xx supercapacitors, when a transmission is sent the storage voltage drops significantly: by approximately 0.24 V. The low series resistance of the Cap-xx supercapacitor is shown from the absence of the spikes on the green trace. Since the sensor will not operate if the voltage drops below 2.5 V, the Panasonic supercapacitor can only be discharged to 2.74 V, whereas the Cap-xx supercapacitor can still function down to 2.5 V. This increases the available energy that can be extracted from the Cap-xx supercapacitor in this application.

This 3-hour period and the measured leaf node power consumption can be used to clarify the evaluation given in Section 2.5. To simplify this evaluation an assumption was made that the node spends half of its operation time below the lower threshold, consuming 0.91 mW. For the charge rates demonstrated an extrapolation was made to cover a 24 hour period, Table 2 shows the estimated charge time required to supply the energy needs of each node in the network for a 24-hour period. It can be seen that SCNode2 which is positioned closest to the transmitter, requires the least charge time due to the higher received power at this distance followed by BATNode1 and SCNode1. As discussed in Section 2.4, the intermittent charge condition for this network (μ_SCNode1_ + μ_SCNode2_ + μ_BATNode1_ < 1) is met (Table 2) and Equation (6) is satisfied for each leaf node in the network. The 1.5 hour shut down time, indicated in Table 2, could be used to power a node at a very short distance or low operating power requirements. Alternatively it could be used to ensure that no nodes in the system will power down.

In summary, this clearly demonstrates that the system has the ability to supply power to many sensors at short range or fewer sensors at a longer range. Overall, a system that supplies a small number of nodes at short range may have niche applications, for example animal tracking by situating a charge station near feeding or water sources. The ability to increase the range will allow for an expansion of applications. Range can be increased by reducing the power consumption of the leaf nodes. For instance, the sensors discussed in [7,8] are able to operate on 2 μW to 2 mW; if the operation is on the lower end of the scale, from Figure 6(a) and along with the circuit efficiency, the system should be able to operate at more than 3 meters. Additionally, reducing the node power consumption reduces the value of μ which means more nodes can be powered for the same distances above. The range of the system can also be increased by increasing the efficiency of the harvesting circuit to higher than 39.1%, *i.e.*, through full wave rectification. Finally, range can be increased by increasing the gain of the transmitting antenna resulting in a narrower beam and less power being transmitted to locations where no nodes are situated.

## 4. Conclusions

A system utilising the sensor's processor power and minimal number of components has been designed that will allow a network of sensor nodes be tracked and powered by means of radio frequency power transfer, allowing indefinite operation of the sensor network. To do this a harvesting/rectifying circuit exhibiting good conversion efficiency (39.1%) has been implemented. The charging circuit impedance, as battery or supercapacitor voltage changes, has been characterised demonstrating a maximum S11 value of −31.5 dB at the tuned input parameters. The near field CST simulated results obtained for the power transfer has shown good agreement with measurements and confirmed that sufficient power can be transferred in an indoor application. It was also demonstrated that the sense voltage output of the harvesting circuit along with the seek protocol can be used to determine if RF power is being received and used as a method of tracking the position of the sensor nodes.

Results showed that a network containing three leaf nodes has been continuously powered by the system. This demonstrated that sufficient power for these three nodes could be supplied. The issues in supplying the energy needs from a controllable intermittent energy source have been discussed such as the need to priorities one sensor over another and to apply additional protocols to deal with this.

Included in this examination is a comparison of two commercially available supercapacitors; it has been noted that the high series resistance, 100 Ω, of the Panasonic supercapacitors reduces the available energy when high currents are drawn from the supercapacitor.

## Figures and Tables

**Table 1.** Leaf node configurations including storage types and voltage thresholds.

**Sensor Designation**	**Storage Type and Manufacture**	**Model**	**Capacity and Series Resistance**	**Lower Trigger Voltage**	**Upper Triggervoltage**	**B-Antenna to L-Antenna Separation Distance** **(cm)**
**SCNode1**	Cap-xx Supercapacitor	GS 280	2 × 0.9F (30 mΩ each) [12]	3.95 V	4.3 V	130 cm
**SCNode2**	Panasonic Supercapacitor	EECF5R5H105	2 × 1F (100 Ω each) [13]	3.95 V	4.3 V	110 cm
**BATNode1**	VARTA NiMH Battery	55602303013	70 mAh [14]	3.75 V	3.9V	120 cm

**Table 2.** Break down of charge time required over 24 hours.

**SensorDesignation**	**Charge Time Requiredin 24 Hours**	**IntermittencyFactor μ**
**SCNode1**	9.03	0.38
**SCNode2**	4.97	0.21
**BATNode1**	8.49	0.35
**Shut down time**	1.51	0.06
